# Supplementary material for: Measuring novice-expert sense of place for a far-away place: Implications for geoscience instruction
Source: PLoS One. 2023 Oct 26;18(10):e0293003. doi: 10.1371/journal.pone.0293003 (PMC10602358; doi:10.1371/journal.pone.0293003)
Supplement: S2 File — (DOCX) [file pone.0293003.s002.docx]

**Supporting information 1 (S1): Survey Instrument**

Which of the following best describes you? Check all that apply:

- Currently living/previously lived in Greenland
- Visited Greenland for personal reasons
- Visited Greenland for professional
- Have never been to Greenland
- Other relationship to Greenland (please describe):

What words would you use to describe Greenland?

In no more than two sentences, how do you personally feel about Greenland?

What is your age?

- <20 years old
- 20-29
- 30-39
- 40-49
- 50-59
- 60-69
- 70+

What is your profession?

**Supporting information 2 (S2): Data File**

The attached Excel file includes the deidentified data that was collected as part of this study separated in Novice and Expert data on different tabs.

**Supporting information 2: Coding Scheme**

| Code | Description | Examples |
| --- | --- | --- |
| Code 1 Overall Impressions | | |
| Positive | Survey respondent has feelings of attachment/admiration for Greenland/the people who live there indicating an overall positive impression of Greenland. | "I have so enjoyed working and traveling in Greenland. The people are friendly and respectful. The land is gentle." |
| Negative | Survey respondent has negative feelings toward Greenland and/or its political or social systems indicating an overall negative impression of Greenland. | "I feel like Greenland is perpetually being purchased. The United States recently tried to buy it, other countries have tried buying it in the past." |
| Neutral | Survey respondent has little to no feelings about Greenland indicating a neutral impression of Greenland. | "No feelings at all, just neutral. I know that Iceland is green and Greenland is icy." |
| Contradicting | Survey respondent has contradicting feelings about Greenland and/or its people and social systems indicating an overall contradictory impression of Greenland. | "I feel conflicted; it is an immense and beautiful landscape and deeply affected by changing climate. It's also a land with a deep history of harmful colonization and the science we've done there can perpetuate that." |
| Code 2 Environment Observations/Associations | | |
| Beautiful/Magical/Special Natural Environment | Survey respondent comments indicate observations or associations relating to Greenland's beautiful, magic, or special natural environment. | "Greenland seems like a beautiful, frozen paradise. I would love to visit to see the beaches and mountains and incredible landscapes." |
| Remote/Isolated/Challenging/Wild/Ice Covered Natural Environment | Survey respondent comments indicate observations or associations relating to Greenland's remote, isolated, challenging, wild, or ice covered natural environment. | "I have never been or don't know anyone who has been, so I know know much about it. I just think of I as empty, cold and icy." |
| Observations surrounding Greenland’s People/Systems/Built Environment | Survey respondent comments indicate observations or associations relating to Greenland's people, systems, or built environment. | "It was my first Arctic experience and changed the course of my life. First exposure to Inuit culture which has also impacted me substantially" |
| Code 3 Concern for Greenland | | |
| Environmental/Scientific Concern | Survey respondent has feelings of concern (worry, fear, etc.) for Greenland's environmental well-being or from a scientific standpoint. | "Having only been once, I feel quite protective about the purity of Greenland. It is. stunning and wild place which really magnifies the impact of the environmental challenge that the world is facing." |
| Cultural/Social/Political Concern | Survey respondent has feelings of concern (worry, fear, etc.) for Greenland's cultural, social, and political well-being. | Terrified for the future of Greenland's people and environment. Desperate to return, desperate to help." |
| Code 4 Greenland’s Importance | | |
| Environment/Scientific Importance | Survey respondent comments indicate Greenland as environmentally or scientifically important. | “I feel that Greenland is an important place and deserves to be taken care of and treated sustainably." |
| Cultural/Social/Political Importance | Survey respondent comments indicate Greenland as culturally, socially, or politically important. | "It is the best run and organized of all arctic countries. Could be a model for other arctic places." |
| Code 5 Interest in Greenland | | |
| Environment/Scientific Interest | Survey respondent comments indicate Greenland as environmentally or scientifically interesting. | "I feel like it's a beautiful place that has much to offer in regards to science. It's a place to study and to marvel, as long as the weather behaves." |
| Cultural/Social/Political Interest | Survey respondent comments indicate Greenland as culturally, socially, or politically interesting. | "Seems like a beautiful country with lots of untouched spectacle. I would like to learn more about the history and culture." |
| Code 6 Feelings of Connection or Attachment to Greenland | | |
| Environment/Scientific Connection | Survey respondent has feelings of connection, attachment, or responsibility for Greenland (e.g. feeling privilege to have been in the place, memories of field work, etc.) | "Greenland provided me with a new insight and experience. Made me more sensitive to Nature." |
| Cultural/Social/Political Connection | Survey respondent has feelings of connection, attachment, or responsibility to the people/culture (e.g. admiration for the people and their communities) | "I love Greenland and feel really lucky to be able to work and visit there. I worry about the ice sheet and also hope that Greenlanders will be able to adapt to climate while maintaining culture and improving equity." |
| Code 7 Greenland Awareness Level | | |
| No Awareness | Survey respondent has no awareness about Greenland. | "I don't feel anything towards Greenland nor do I even know what goes on in Greenland" |
| Basic | Survey respondent has only general knowledge about Greenland’s natural environment, social or political systems, or culture. | "A unique ecosystem, important as a polar region, storing carbon and such." |
| Developing | Survey respondent knows more advanced information about Greenland’s natural environment, social and political systems, and/or culture. | "Greenland is a valuable asset for scientific and polar research. Also that it is a very cold and remote location that offers untouched beauty." |
| Complex | Survey respondent knows a considerable amount about Greenland’s natural environment, social and political systems, and culture. | "I personally am fascinated, horrified, and saddened by Greenland. Fascinated by its intense climates and geography, horrified by its harsh environment, and saddened by its perfect example of a landscape we are actively destroying." |
