# Supplementary material for: Measuring novice-expert sense of place for a far-away place: Implications for geoscience instruction
Source: PLoS One. 2023 Oct 26;18(10):e0293003. doi: 10.1371/journal.pone.0293003 (PMC10602358; doi:10.1371/journal.pone.0293003)
Supplement: Attachment — Submitted filename: ResponseToReviewer.docx [file pone.0293003.s003.docx]

**Response to Editor and Reviewer Comments and Requests**

**Editor Request:** Please ensure that your manuscript meets PLOS ONE's style requirements, including those for file naming.

**How we addressed it:** We updated the file formatting, and it now meets the PLOS One style requirements.

**Editor Request:** Please amend your current ethics statement to address the following concerns:
a) Did participants provide their written or verbal informed consent to participate in this study?
b) If consent was verbal, please explain i) why written consent was not obtained, ii) how you documented participant consent, and iii) whether the ethics committees/IRB approved this consent procedure.

**How we addressed it:** In our study, we collected electronic consent from all survey respondents. We added the following sentence to the ethics statement (located now within Methodology, subsection Survey Instrument: *All participants provided electronic consent to participate in the study.*

**Editor Request:** We note that the grant information you provided in the ‘Funding Information’ and ‘Financial Disclosure’ sections do not match. When you resubmit, please ensure that you provide the correct grant numbers for the awards you received for your study in the ‘Funding Information’ section.

Thank you for stating the following in the Acknowledgments Section of your manuscript: 
   "The research was funded as part of the PolarPASS project by the National Science Foundation under awards OPP-2021503, OPP-2021275 and OPP-2021543 and National Oceanic and Atmospheric Administration through award NA17OAR4320101. We are grateful for contributions from Lynne Harden on the research design and discussions with Steven Semken on the framing. Meghan Henderson wrote the Excel Macros that allowed an easy lookup of words in the lexica. Ami Nacu-Schmidt created the illustration in figure 1. We received IRB approval for this study from the University of Colorado IRB office under protocol 21-0189."

We note that you have provided funding information that is not currently declared in your Funding Statement. However, funding information should not appear in the Acknowledgments section or other areas of your manuscript. We will only publish funding information present in the Funding Statement section of the online submission form. 
Please remove any funding-related text from the manuscript and let us know how you would like to update your Funding Statement. Currently, your Funding Statement reads as follows: 
 "OPP-2021503
OPP-2021275
OPP-2021543"

Please include your amended statements within your cover letter; we will change the online submission form on your behalf.

**How to address – our response:**

We removed the funding information from the Acknowledgement section. The funding information and role of the funder is now added to the coverletter. It should read as follows:

“*National Science Foundation awards OPP-2021503, OPP-2021275 and OPP-2021543 
National Oceanic and Atmospheric Administration award NA17OAR4320101. The funders had no role in study design, data collection and analysis, decision to publish, or preparation of the manuscript*.”

**Editor Request:** In your Data Availability statement, you have not specified where the minimal data set underlying the results described in your manuscript can be found. PLOS defines a study's minimal data set as the underlying data used to reach the conclusions drawn in the manuscript and any additional data required to replicate the reported study findings in their entirety. All PLOS journals require that the minimal data set be made fully available. For more information about our data policy, please see <http://journals.plos.org/plosone/s/data-availability>.

"Upon re-submitting your revised manuscript, please upload your study’s minimal underlying data set as either Supporting Information files or to a stable, public repository and include the relevant URLs, DOIs, or accession numbers within your revised cover letter. For a list of acceptable repositories, please see <http://journals.plos.org/plosone/s/data-availability#loc-recommended-repositories>. Any potentially identifying patient information must be fully anonymized.

Important: If there are ethical or legal restrictions to sharing your data publicly, please explain these restrictions in detail. Please see our guidelines for more information on what we consider unacceptable restrictions to publicly sharing data: <http://journals.plos.org/plosone/s/data-availability#loc-unacceptable-data-access-restrictions>. Note that it is not acceptable for the authors to be the sole named individuals responsible for ensuring data access.

We will update your Data Availability statement to reflect the information you provide in your cover letter.

**How we addressed it:** We have uploaded a file that includes all the data to replicate the study. All data is anonymous and not identifiable.

**Editor Request:** Please ensure that you include a title page within your main document. You should list all authors and all affiliations as per our author instructions and clearly indicate the corresponding author.

**How we addressed it:** We have reformatted the title page to match the PLOS One guidelines: Title page: List title, authors, and affiliations as the first page of the manuscript. We have now clearly indicated the corresponding author. Note that our long title does not exceed the character limit of the short title. We, therefore, suggest using the title both as the long and the short title. If you prefer to have a short title that is shorter than the long title please use as the short title: “*Measuring novice-expert sense of place for a far-away place*”.

**Editor Request:** Your ethics statement should only appear in the Methods section of your manuscript. If your ethics statement is written in any section besides the Methods, please move it to the Methods section and delete it from any other section. Please ensure that your ethics statement is included in your manuscript, as the ethics statement entered into the online submission form will not be published alongside your manuscript.

**How we addressed it:** We moved the ethics statement to the sub-section “Survey instrument” in the “Methodology” section

[Note: HTML markup is below. Please do not edit.]

Reviewers' comments:

Reviewer's Responses to Questions

**Comments to the Author**

1. Is the manuscript technically sound, and do the data support the conclusions?

The manuscript must describe a technically sound piece of scientific research with data that supports the conclusions. Experiments must have been conducted rigorously, with appropriate controls, replication, and sample sizes. The conclusions must be drawn appropriately based on the data presented.

Reviewer #1: Yes

2. Has the statistical analysis been performed appropriately and rigorously?

Reviewer #1: Yes

3. Have the authors made all data underlying the findings in their manuscript fully available?

The [PLOS Data policy](http://www.plosone.org/static/policies.action#sharing) requires authors to make all data underlying the findings described in their manuscript fully available without restriction, with rare exception (please refer to the Data Availability Statement in the manuscript PDF file). The data should be provided as part of the manuscript or its supporting information, or deposited to a public repository. For example, in addition to summary statistics, the data points behind means, medians and variance measures should be available. If there are restrictions on publicly sharing data—e.g. participant privacy or use of data from a third party—those must be specified.

Reviewer #1: Yes

4. Is the manuscript presented in an intelligible fashion and written in standard English?

PLOS ONE does not copyedit accepted manuscripts, so the language in submitted articles must be clear, correct, and unambiguous. Any typographical or grammatical errors should be corrected at revision, so please note any specific errors here.

Reviewer #1: Yes

5. Review Comments to the Author

Please use the space provided to explain your answers to the questions above. You may also include additional comments for the author, including concerns about dual publication, research ethics, or publication ethics. (Please upload your review as an attachment if it exceeds 20,000 characters)

Reviewer #1: I thought this was a very well-organized, readable manuscript. I learned a great deal about the methodology of using this approach to analyzing sense of place. The only questions I had about the manuscript were the limitations imposed by the two groups--novice and expert--and how these groups were defined. The novice group was mostly young, from a research university. No racial or ethnic demographics were provided. The expert group was highly-educated, older, more experienced. So I wondered first if the novice group had been made up of faculty and other older professionals would they have responded more closely to the expert group? Undergraduates might have a less nuanced understanding of the cultural and geopolitical aspects of a place they've never been than university faculty would, regardless of whether they had been there. Likewise, if the novice group had included students from indigenous populations in the U.S., they might have a very different way of responding than the U Colorado Boulder students.

I thought the limitation of two sentences for the first question in your survey might actually support a less nuanced picture. What would have changed in your research if you had left the amount they wrote open-ended? Some people think while they write so they might reach some of the more complex issues if they were expected to write more. They might reflect more on what they have heard or know about a place rather than hitting on their first impression (cold, remote, beautiful, melting).

Another thought I had was that building knowledge or culture and other aspects of a place is important, but how will you distinguish in your follow-up research whether it's the virtual experience or the type of content that is the most significant in understanding place. For example, I could see where incorporating videos of various people who live in Greenland into your virtual field trip might influence students' perception of that place. Adding readings (novels or articles) that highlight this content might be equally useful. I think you will need to be very careful in distinguishing whether it's the media or the message that matters. I have been very impacted by just meeting one other person from a place I have never been, which can be very influential to how you understand a place. Incorporating a virtual talk from someone who lives there, possibly from the indigenous culture or a faculty member, could be very influential on what someone knows about a place and how they feel about it.

Overall I thought this was extremely well-written and very thought provoking. Thanks so much for providing me the opportunity to review.

6. PLOS authors have the option to publish the peer review history of their article ([what does this mean?](https://journals.plos.org/plosone/s/editorial-and-peer-review-process#loc-peer-review-history)). If published, this will include your full peer review and any attached files.


If you choose “no”, your identity will remain anonymous but your review may still be made public.


**Do you want your identity to be public for this peer review?** For information about this choice, including consent withdrawal, please see our [Privacy Policy](https://www.plos.org/privacy-policy).

Reviewer #1: **Yes:** Diana M Dalbotten

**How did we address reviewer comments:**

1) Limitations of study

Your point is well-taken. The make-up of the pool of respondents is of course very important to the results. The reason why we chose the undergraduate college student as our novice population is that this is a baseline study as part of a larger study in which we implement immersive experiences in undergraduate classrooms. For the follow-on study, we have recruited undergraduate students from a diversity of schools (including students with Indigenous backgrounds and students with a background that is traditionally underrepresented in STEM disciplines). For this study, we did not ask respondents about their racial or ethnic identity.

To address this point in the manuscript, we have added the following text to the Limitations section: *”We did not collect demographic data beyond age and profession, and are therefore unable to report ethnic and racial backgrounds of the respondents. Respondents with a racial or ethnic background that is grounded in strong place-meaning (e.g., Indigenous or Native), may show a stronger place meaning or attachment. We focused our recruitment on the Greenland research community. Our results may look different, had we recruited only Greenlandic residents, likely showing an even stronger place attachment.”*

In addition, we have added the following test to the Study Participant section*: “We selected U.S. undergraduate college students as our novice population because very few students at a U.S. college would have visited Greenland before, thus, responding to the survey with the expertise of a novice. The reason why we surveyed undergraduate students is because this study is part of a larger study that aims to improve undergraduate college instruction using immersive virtual field experiences and therefore, responses from college students provide a meaningful baseline for novices*.” [and further on in the same paragraph, we added about the experts] “*These networks include many people who are conducting research in Greenland, live in Greenland or spent time in Greenland, and thus provide access to people who have visited, worked, lived or currently live in Greenland*.”

2) Changes in the way open-ended question was asked

The reason why we limited the writing to two sentences is that we wanted to capture what first comes to mind for the respondents. We further wanted to keep the survey short and be able to compare the results of different respondents. Had we left the question without guidance on length, one respondent would have written 10 sentences, while others may have written just one sentence. Providing guidance to keep the answers short allowed for responses that were comparable between all respondents.

To address this point in the manuscript, we have added the following sentence to the Methodology section: “*In asking respondents to keep their answer to two sentences, we forced sharing of the initial associations with the place and allowed for comparable response length between study subjects.*“

3) Follow on research limitation:

This is a very good point and helpful advice. We will keep this in mind for our future study design.

[NOTE: If reviewer comments were submitted as an attachment file, they will be attached to this email and accessible via the submission site. Please log into your account, locate the manuscript record, and check for the action link "View Attachments". If this link does not appear, there are no attachment files.]

While revising your submission, please upload your figure files to the Preflight Analysis and Conversion Engine (PACE) digital diagnostic tool, <https://pacev2.apexcovantage.com/>. PACE helps ensure that figures meet PLOS requirements. To use PACE, you must first register as a user. Registration is free. Then, login and navigate to the UPLOAD tab, where you will find detailed instructions on how to use the tool. If you encounter any issues or have any questions when using PACE, please email PLOS at [figures@plos.org](mailto:figures@plos.org). Please note that Supporting Information files do not need this step.

**How we addressed this:** We uploaded all four figures to PACE and they look clear. See screenshot below.
