# Supplementary material for: Measuring novice-expert sense of place for a far-away place: Implications for geoscience instruction
Source: PLoS One. 2023 Oct 26;18(10):e0293003. doi: 10.1371/journal.pone.0293003 (PMC10602358; doi:10.1371/journal.pone.0293003)
Supplement: Attachment — Submitted filename: Response to Reviewers.docx [file pone.0293003.s004.docx]

Response to Reviewers

**Reviewer and Editor Comments:**

Reviewer 2 pointed out the need for reframing the hypothesis to clearly elucidate the role of the virtual experience in the context of measuring novice-expert sense of place. This is a valid concern and addressing it will greatly enhance the overall contribution of your paper. To this end, I suggest making the following revisions:

Introduction:

a. Clearly state the objective of your study, emphasizing the investigation of how virtual experiences contribute to measuring novice-expert sense of place for a far-away location.

b. Provide a brief overview of the existing literature on the use of virtual experiences in geoscience instruction and their potential impacts on developing a sense of place.

Hypothesis:

a. Revisit your hypothesis and reframe it to explicitly incorporate (or remove the mention of) the role of the virtual experience. Consider highlighting how the virtual experience affects novice-expert sense of place formation and whether it influences participants' spatial understanding, emotional connection, or both.

b. Justify the need for investigating the impact of the virtual experience, particularly in the context of assessing sense of place for a far-away location where physical visits may be limited.

Results and Discussion:

a. Analyze and interpret the data collected in the context of the reframed hypothesis, emphasizing the relationship between the virtual experience and participants' sense of place.

b. Discuss any limitations or challenges associated with using a virtual experience as a proxy for a physical visit, and address how these limitations may impact the interpretation of the results.

**How did we address the comment:**

The concern that reviewer 2 raised is valid and we appreciate you laying out how to address the concern. We decided to follow your recommendation and remove the mention of virtual field trips from the hypothesis and framing of the paper and instead we mention that the study may have implications on virtual field trips. All changes are provided in a version that includes “track changes”. Specifically we changed:

**Abstract** – removed mention of virtual field trips in the motivation and hypothesis and focused on the research design that compares novices and experts. At the end of the abstract, we rephrased a sentence to the findings may have implications for virtual field trips in education instead of using it as a motivation.

**Introduction** – removed the mention of virtual field trips as part of the motivation and instead rephrased that the study may have implications for virtual field trips in education.

**Results:** No changes since we are not including data around virtual field trips

**Discussion:** We rephrased the text to suggest that future research may explore the impacts on virtual field trips and removed any connection to the motivation of the study.

**Conclusion:** We rephrased the text to remove mention of the virtual field trips as part of the study motivation and instead phrased it to suggest that future research may be needed to stuy the impact on virtual field trips in education.
